# Honest signaling and the double counting of inclusive fitness

**DOI:** 10.1002/evl3.138

**Published:** 2019-09-04

**Authors:** Samuel R. Levin, Shana M. Caro, Ashleigh S. Griffin, Stuart A. West

**Affiliations:** ^1^ Department of Zoology University of Oxford Oxford OX1 3PS United Kingdom; ^2^ Department of Ecology, Evolution and Environmental Biology Columbia University NY 10027 New York

**Keywords:** Begging, double counting, inclusive fitness, parent–offspring conflict, signaling

## Abstract

Inclusive fitness requires a careful accounting of all the fitness effects of a particular behavior. Verbal arguments can potentially exaggerate the inclusive fitness consequences of a behavior by including the fitness of relatives that was not caused by that behavior, leading to error. We show how this “double‐counting” error can arise, with a recent example from the signaling literature. In particular, we examine the recent debate over whether parental divorce increases parent–offspring conflict, selecting for less honest signaling. We found that, when all the inclusive fitness consequences are accounted for, parental divorce increases conflict between siblings, in a way that they can select for less honest signaling. This prediction is consistent with the empirical data. More generally, our results illustrate how verbal arguments can be misleading, emphasizing the advantage of formal mathematical models.

**Impact Summary**

Evolutionary theory predicts that organisms should adopt traits that increase their inclusive fitness, a measure of fitness that includes contributions to relatives, and this idea has been applied to a wide range of empirical scenarios. But knowing how traits actually impact inclusive fitness requires a careful accounting of all the effects of a trait. We discuss a common mistake in calculating inclusive fitness, known as “double counting,” where the effects of a trait are counted twice. We show how this problem can lead to incorrect predictions, and how it can be avoided. We illustrate this potential problem with an analysis of whether divorce should have an impact on how honestly offspring should beg for food. Contrary to the verbal argument in a recent paper, we show that divorce should cause offspring to be less honest when signaling their need because divorce causes future siblings to be only half‐siblings. Our prediction is supported by empirical data across 60 species of birds.

Evolutionary theory predicts that selection for honest signaling can be reduced when there is greater conflict between individuals (Grafen 1990; Maynard Smith and Harpe 2003). This prediction can be difficult to test with studies on single species because the factors that determine conflict may not vary sufficiently to produce detectable variation (Popat et al. 2015). Caro et al. (2016) circumvented this problem with a comparative study across 60 species of birds, and examined whether greater conflict led to a weaker correlation between the intensity with which offspring beg and their long‐term need (less honest signaling). In support of theory, Caro et al. (2016) found that offspring signaled less honestly when (i) they face competition from current siblings, (ii) their parents are more likely to breed again, and (iii) parents are more likely to die or divorce (i.e., change mating partners between breeding bouts).

Bebbington and Kingma (2017) questioned one aspect of the third result with a verbal argument. Caro et al. (2016) argued that divorce should increase parent–offspring conflict because it means that future siblings, produced after the divorce, will only be half‐siblings. This reduction in relatedness between siblings increases parent–offspring conflict (Hamilton 1964; Trivers 1974). In contrast, Bebbington and Kingma (2017) argued that divorce should have no impact on offspring honesty because an individual will gain two sets of half‐siblings, canceling out the effects of losing one set of full siblings. Instead, they suggested a number of alternative hypotheses that could explain the data.

We show here that the correct prediction depends on the specific biological scenario envisioned. Bebbington and Kingma's (2017) argument can be interpreted in a number of ways, but their verbal and graphical argument suggests that they are making what is termed as “double‐counting error” (Grafen 1982, 1984; Queller 1996). When considering the inclusive fitness consequences of divorce, it appears that they are summing across all the siblings that were produced in the future (the white and gray offspring in their fig. 1). This leads to an error because it counts offspring multiple times as part of the fitness of multiple individuals. Instead, when considering the evolution of a trait, we need to focus on the specific consequences of variation in that trait, the "inclusive fitness effect" (Hamilton 1964; Grafen 1982, 1984; Taylor 1989, 1990; Queller 1996; Frank 1998; West et al. 2007). Whether a rare mutant allele will spread to fixation depends on the change in fitness due specifically to that allele, not on the total fitness of the individual and its relatives.

**Figure 1:**
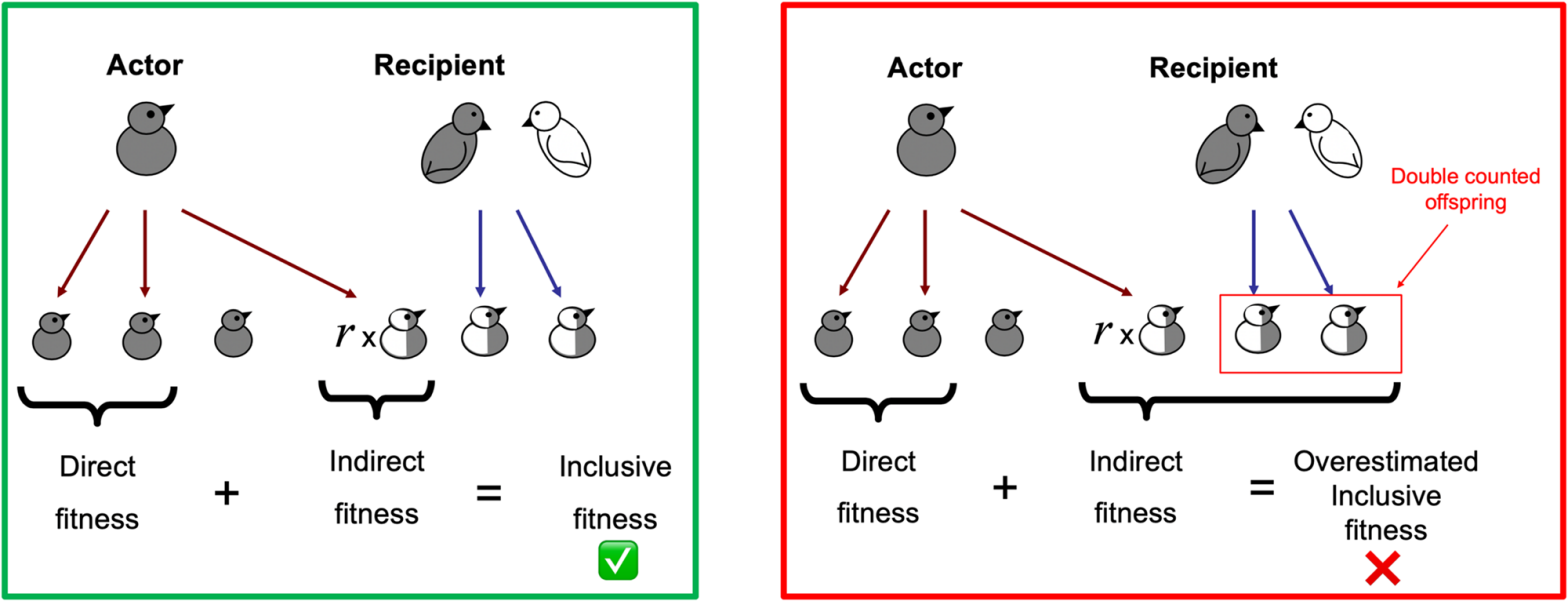
Inclusive fitness requires counting only the fitness effects of the actor (Hamilton 1964). In this case, we are observing only the indirect fitness of the actor, as we focus on its contribution to future sibs. The correct approach is to count only the future sibs that the actor creates by its action (green panel). One form of double counting is to count all of the offspring of the recipient (in this case, a parent), and not just those contributed by the actor (red panel) (Grafen 1982). This figure is adapted from West et al. (2007).

We present a simple inclusive fitness model to show that, under standard assumptions, and without double counting, theory predicts that divorce should matter. We then use a neighbor‐modulated fitness approach to model this case more formally. Finally, we consider empirical support for the alternative hypotheses proposed by Bebbington and Kingma (2017). Our overall aim is to use an analysis of this particular problem to examine more general issues about how problems with fitness accounting can arise from simple verbal arguments.

## Inclusive Fitness and Double Counting

Caro et al. (2016) argued that if the parents of an individual divorce, then that individual will be half as related to future siblings, and so will be selected to obtain more resources in the short term from their parents, through less honest signaling. Bebbington and Kingma (2017) argued that this prediction should not hold because divorce would also lead to that individual having twice as many siblings, because each parent happens to raise a separate brood. Bebbington and Kingma (2017) argued that these two effects, twice as many offspring that are half as related, would exactly cancel, and so individuals should be indifferent to the likelihood that their parents will divorce.

However, what matters in the eyes of selection is the inclusive fitness *effect* of a trait, and not the total number of relatives produced (Hamilton 1964). Inclusive fitness does not include all the offspring produced by relatives, only those that are a result of the behavior of the individual whose fitness we are measuring (Fig. 1, but see also in West et al. 2007, fig. 3; or Davies et al. 2012, box 11.4). For example, if the helping behavior of an actor leads to the beneficiary of that help producing another offspring, then that offspring would be counted in the inclusive fitness of the actor (indirect benefit) but not the beneficiary. To count that offspring both times, or even more if we also considered other relatives, is a form of the double‐counting error (Fig. 1; Grafen 1982; Queller 1996). Bebbington and Kingma (2017) present their verbal argument as two sentences and figure 1 (p. 133) of their paper: “However, we argue that divorce (right‐hand panel [of fig. 1]) does not promote dishonesty in this way because both parents will continue breeding and hence produce two sets of half‐siblings, which together have equal or even higher value than one set of full siblings (total relatedness 2 × 0.25 = 0.5).” This argument appears to make this double‐counting error, by counting all the offspring in the fitness effect (both white and gray offspring in their fig. 1), and not isolating those contributed by the actor.

**Figure 2:**
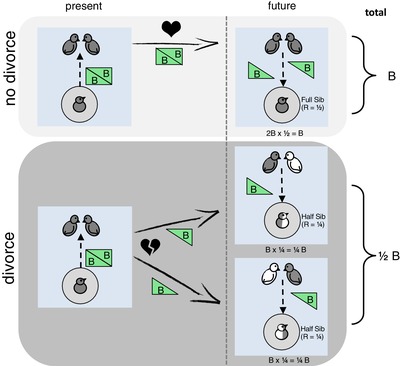
Divorce favors dishonesty because it causes offspring to be less invested in their future siblings. A proper counting of inclusive fitness requires isolating the direct effects of the trait, shown in green. Under monogamy, the effect the offspring has on its parents remains with both parents, and then is doled out to full siblings. In the case of divorce, the effects are divided between separated parents, and then doled out to half‐siblings.

To illustrate this in simple terms, imagine a baby bird that signaled that it needed less food, and hence provided a marginal fitness benefit *B* to each parent, and the parents pass this benefit on future offspring (Fig. 2). Put simply, the parent invests less in the current brood, and more in the future brood. In the case of monogamy, a baby is related to its future (full) siblings by 0.5, these receive a benefit of 2*B* (*B* from each parent), and therefore the total inclusive fitness effect is 2 x *B* x 0.5 = *B*. In the case of divorce, two sets of half‐siblings, related by 0.25, each receive *B*, and the total inclusive fitness effect is 2 x *B* x 0.25 = 0.5*B*. Therefore, divorce leads baby birds in the first brood having less interest, from a fitness perspective, in the future brood, and so would be favored to obtain a greater share of paternal resources, by less honest signaling.

One interpretation of Bebbington and Kingma's (2017) argument is that it would require that the 2*B* given to the original parents translates to 2*B* in *each* of the remarriages. Phrasing the problem in terms of offspring number, without divorce a baby bird gives, for example, an extra offspring to its mother and an extra offspring to its father, which translates to two full siblings (2 × 0.5). Under divorce, this translates to two half‐siblings (2 × 0.25). Bebbington and Kingma's (2017) argument requires that, under divorce, the extra offspring given to mom and dad somehow double. Next, we will return to another possible biological scenario for Bebbington and Kingma's (2017) argument, which is theoretically interesting, but unlikely to apply to signaling in birds.

It is possible that if divorce is common then the other parent may also be contributing an extra *B*, but that does not matter—it is not the consequence of the behavior of the individual whose behavior we are examining. Hamilton (1964) was the first to realize the potential for this confusion, and so he explicitly addressed it in his original definition of inclusive fitness, where he stressed the need to strip all components of fitness “which can be considered as due to the individual's social environment”, and to focus on the “fractions of the quantities of harm and benefit which the individual himself causes.” In the case we are considering, Hamilton's point means multiplying the benefit for future broods, after divorce, by *B* and not 2*B*. The potential for this double‐counting error has been highlighted by Grafen (1982; 1984), Queller (1996), and others (West et al. 2007; Davies et al. 2012).

To provide another way of thinking about this problem, instead of a baby bird providing a benefit *B* to their parent, we can think of the baby bird as reducing the parents’ overall resources. Each parent starts with *V* resources, and fitness is a function of the average resources of the parents. A baby can reduce its parents’ total resources by some fraction, *f* (0 < *f* < 1), such that they enter the next breeding season with ((1) − f)*V* resources. Assuming these resources are taken *equally* from each parent, a baby takes *fV*/2 resources from each parent. In the case of monogamy, a baby takes *fV*/2 + *fV*/2 = *fV* from its full siblings (*r* = 0.5), and therefore the effect is −0.5*fV*. In the case of divorce, a baby take *fV*/2 from each of its two half‐sibling broods (*r* = 0.25), such that the total effect is −0.25*fV*. From an inclusive fitness perspective, using up resources has a smaller negative inclusive fitness effect in the case of divorce.

## A Neighbor‐Modulated Fitness Model

Inclusive fitness theory requires a careful accounting of all the fitness effects of a particular behavior, which can be complicated when reasoning verbally (Taylor and Frank 1996; Frank 1998; Taylor et al. 2007; Gardner et al. 2011). As illustrated above, and by previous discussions of the double‐counting error, this could lead to behavioral consequences being incorrectly added or missed (Grafen 1982; Queller 1996). A solution to this is to develop theory with the neighbor‐modulated fitness method of Taylor and Frank (1996), Frank (1997, 1998), Rousset (2004), and Taylor et al. (2007), which provides a powerful and relatively simple way to derive an expression for the fitness consequences of a behavior.

We use the neighbor‐modulated fitness method to theoretically examine whether the potential for divorce should influence the behavior of an offspring. We take a Maynard Smith (1991) approach, and deliberately develop a very simple model, to illustrate the general point in an accessible way, rather than a more complicated signaling model that would be less easy to follow. As such, we assume that signaling is optimal, and focus only on the marginal fitness effect of divorce.

We assume that there are only two years of breeding. There is a probability *d* that parents “divorce” between these two years, in which case they pair up with another divorced parent in their second year. We assume that an offspring in the first year of breeding can extract a proportion *f* of its parents’ total resources, and that parents give the remaining (1−f) of their resources to offspring in the second year. We wish to find if the amount of resources that the offspring should extract in their first year, *f*, is influenced by the divorce rate *d*.

The fitness, *w*, of an individual is a function of its own strategy (*f*), the strategy of its full sibling, $F_{full}$, the strategy of its half‐sibling, $F_{half}$, and the population wide average, $F_{pop}\;$ is

(1) $$\begin{array}{ccc} & & w\left(f,\;F_{\mathrm{full}},\;F_{\mathrm{half}},\;F_{\mathrm{pop}}\right)=f\left(\left(1-d\right)\left(1-F_{\mathrm{full}}\right)\right.\\ & & \;\left.+\;d\left(\frac{\left(\left(1-F_{\mathrm{half}}\right)+\;\left(1-F_{\mathrm{pop}}\right)\right)}{2}\right)\right)\;. \end{array}$$

We wish to find the evolutionarily stable strategy (ESS), which is the strategy that cannot be beaten by any other strategy, and so would be stable under natural selection (Maynard Smith and Price 1973). We assume that relatedness is equal to 1/2, 1/4, and 0, for full siblings, half‐siblings, and a random member of the population, respectively. Using Taylor and Frank's (1996) methodology, we find that the ESS is

(2) $$f^{*}=\frac{8}{3(4-d)}.$$

**Figure 3:**
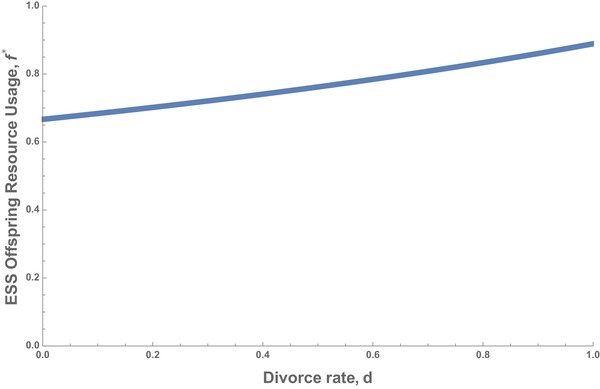
Divorce increases the optimal level of resources an offspring should take from its parents, a proxy for honesty of signaling (eq. (2)).

In this case, divorce always matters, with increasing divorce rate causing babies to take more resources from their parents (Fig. 3). This result formalizes Caro et al.’s (2016) prediction that a greater likelihood of divorce leads to offspring being favored to extract more resources from their parents, and therefore being selected to signal less honestly.

## The Real World

Caro et al. (2016) found that offspring signaled less honestly when their parents were likely to divorce or die. They combined data from divorce and death because they shared a theoretical basis, with both leading to future offspring produced by parents being half‐siblings. Furthermore, in that dataset, there was no significant difference between the influence of divorce and death.

Based on their argument that divorce rates should not matter, Bebbington and Kingma (2017) proposed three alternative mechanisms that might be driving the patterns found by Caro et al. (2016), which they admit are speculative:

(1) Pair bond duration could be confounded by clutch size and offspring competition. This is a valid concern, as brood size and the likelihood of parents breeding together again are correlated. However, Caro et al. (2016) specifically accounted for this by controlling for brood size in their analyses. Furthermore, we tested for collinearity by calculating variance inflation factors for Caro et al.'s 2016 model, and found low VIF values for all fixed effects well below the established cutoff of 10, or the more stringent cutoff of 3 (brood size VIF: 1.83; future reproduction VIF: 2.14; full sibling vs. half‐sibling VIF: 2.44; Zuur et al. 2010; Montgomery et al. 2012). This indicates that brood size did not confound Caro et al.’s 2016 analyses.
(2) Divorce could be linked to competition for mates, making offspring dishonesty the result of higher levels of competitiveness in adults. This is an interesting and plausible hypothesis, but there are no data yet to support it. Further, even if adult competitiveness was correlated with offspring competitiveness as a result of pleiotropy, selective pressures (e.g., divorce) acting on juvenile competitiveness should still shape behavior, and we would still expect the qualitative differences in honesty predicted by Caro et al. (2016). More generally there is no theoretical reason to expect adults and offspring to be incapable of behaving differently at different times in their life. The analogous suggestion that offspring and adult behavior would have to be correlated was an incorrect criticism of parent–offspring conflict theory (Trivers 1974; Dawkins 1976; Godfray 1995).
(3) Divorce could be linked to parents’ investing less in their current offspring because short‐term pair bonds raise conflict between parents. Scramble competition refers to competition between siblings (begging as direct competition over access to parents rather than begging as a “signal”). This is not an alternative to kin selection, as the influence it has on signaling is driven by an analogous decrease in relatedness to their partner's future offspring (Parker and Mock 1998). If conflict reduces the resources parents provide to offspring, this should enhance the effect of divorce on offspring dishonesty. Furthermore, even if this occurred, the effect would be small relative to the halving of relatedness cause by divorce.

To summarize, the empirical data support Caro et al.’s (2016) hypothesis, but not the alternatives suggested by Bebbington and Kingma (2017). We are not suggesting that divorce will be the only factor that matters, but rather that its influence is large enough to be detected in an across species study, where other factors are also varying. This emphasizes the need to consider the effect sizes of alternative explanations. Given that divorce decreases the relative value of future siblings by half, alternative explanations would need equally strong selective pressures to outweigh this influence.

## Future Extensions

Our above model was an idealized simplification, which aimed to illustrate the point that in the simplest case, divorce matters for offspring behavior. There are a number of ways in which this model could be elaborated, to provide more specific predictions for scenarios of particular empirical interest. For example: the effects of signaling on parents’ resources could be multiplicative, not additive; the effects on each parent might differ, potentially leading to intragenomic conflict; likelihood of divorce could vary with parental quality; or divorced parents might not breed again, or might breed with a lower quality individual. Another possibility is that death can have a more complicated influence than divorce because the death of one parent halves both relatedness to and the number of future siblings.

A possible biological interpretation of Bebbington and Kingma's (2017) argument was pointed out to us (A. Gardner, pers. comm.), which could lead to divorce having no influence. If the fitness consequence of offspring signaling need less is to give each parent a fixed number of additional *successful gametes*, then divorce should not matter. However, this argument requires that the factor limiting reproduction is the ability to produce gametes—this is not the case in birds, where the cost of feeding young dominates the cost of reproduction (Peterson et al. 1990; Visser and Lessells 2001). In addition: (1) if it is only the male or the female gametes that are limiting, then divorce should still matter because under monogamy both parents get extra fertilized gametes; (2) if both sexes are limiting in gamete number, divorce still matters because under divorce the extra gametes given to parents do not get fertilized. Thus, the word “successful” in the term “successful gametes” appears to contain some nonlinearity in fitness effects, discussed above. Again, this emphasizes the need for formal models of biologically relevant scenarios.

Bebbington and Kingma (2017) considered another possible extension, in which divorce raises the fitness of parents, following a comparative study by Culina et al. (2015). To eliminate the effect of divorce on offspring honesty, divorce would have to at least double the fitness of divorced parents (see our modeling section). In contrast to this, Culina et al. (2015) found that divorce increased fitness by an average of only 37% more nestlings or fledglings, in a representative sample of 15 species from that analysis with data on the number of offspring produced before and after divorce. We investigated the possibility that the fitness consequences of divorce might eliminate the effect of divorce on honesty with an exploratory analysis on 15 species where there are data on both honesty and the fitness consequences of divorce. We found that, even when taking fitness consequences into account, and with a much smaller sample size, divorce still had a significant effect on offspring honesty (pMCMC = 0.0476*, *n* = 15 species; MCMCglmm model includes phylogeny, study, species, brood size, future reproduction, the fitness consequence of divorce, and the likelihood of divorce and/or parental death). Nonetheless, Bebbington and Kingma (2017) have raised an interesting additional factor, and future work could test the role of fitness consequences of divorce more thoroughly, exploring additional factors.

## Conclusions

To conclude, we suggest two take home messages regarding the application of inclusive fitness theory to specific biological cases. First, care must be taken with fitness accounting when formulating verbal predictions. Formal theoretical models can help resolve ambiguities and clarify predictions. Second, progress can be hindered when alternative mechanisms or additional factors are mistaken for competing hypotheses. For example, the distinction between scramble competition and kin selection is a false one, as the former rests in part on the latter. Future progress is likely to be maximized by the interplay between theory and data.

Associate Editor: A. Gardner
